# Histamine N-methyltransferase Modulates Human Bronchial Smooth Muscle Contraction

**DOI:** 10.1155/S0962935194000153

**Published:** 1994

**Authors:** J. Tamaoki, A. Chiyotani, E. Tagaya, K. Isono, K. Konno

**Affiliations:** First Department of Medicine Tokyo Women's Medical College Tokyo 162 Japan

## Abstract

To elucidate the modulatory role of histamine-degrading enzymes in airway constrictor responses, human bronchial strips were studied under isometric conditions *in vitro*. Pretreatment of tissues with the histamine N-methyltransferase (HMT) inhibitor SKF 91488 specifically potentiated the contractile responses to histamine, causing a leftward displacement of the concentration response curves, whereas the diamine oxidase inhibitor aminoguanidine had no effect. This potentiation was attenuated by mechanical removal of the epithelium. The HMT activity was detected in the human bronchi, which was less in the epithelium-denuded tissues than the epithelium-intact tissues. These results suggest that HMT localized to the airway epithelium may play a protective role against histamine-mediated bronchoconstriction in humans.


Mediators of Inflammation 3, 125–129 (1994)

### Histamine N-methyltransferase modulates human bronchial smooth muscle contraction

J. Tamaoki,[CA] A. Chiyotani, E. Tagaya, K. Isono and K. Konno

First Department of Medicine, Tokyo Women's Medical College, Tokyo 162, Japan

[CA] Corresponding Author

**To elucidate the modulatory role of histamine-degrading enzymes in airway constrictor responses, human bronchial strips were studied under isometric conditions *in vitro*. Pretreatment of tissues with the histamine N-methyltransferase (HMT) inhibitor SKF 91488 specifically potentiated the contractile responses to histamine, causing a leftward displacement of the concentration–response curves, whereas the diamine oxidase inhibitor aminoguanidine had no effect. This potentiation was attenuated by mechanical removal of the epithelium. The HMT activity was detected in the human bronchi, which was less in the epithelium-denuded tissues than the epithelium-intact tissues. These results suggest that HMT localized to the airway epithelium may play a protective role against histamine-mediated bronchoconstriction in humans.**

**Key words:** Asthma, Bronchial smooth muscle contraction, Histamine N-methyltransferase

### Introduction

Histamine is released from mast cells and basophils by a variety of stimuli including antigen cross-linked IgE, and induces airway smooth muscle contraction, microvascular leakage[1] and mucus production.[2] Thus histamine probably plays a principal role in the pathogenesis of immediate asthmatic responses. In addition, because increased bronchial responsiveness to histamine is a well-characterized feature of asthma, bronchial provocation by histamine has routinely been used to facilitate its diagnosis.[3]

It has been known that histamine can be metabolized by two major pathways in the body;[4,5] 50 to 70% of histamine is metabolized by histamine N-methyltransferase (HMT, EC 2.1.1.8), located in the small intestine, liver, kidney and leukocytes, into N-methylhistamine, and the remaining 30 to 45% is metabolized by diamine oxidase (DAO, EC 1.4.3.6), also called histaminase, located in intestinal mucosa, placenta, liver, skin, kidney, neutrophils and eosinophils, to imidazole acetic acid. Recent studies on guinea-pig trachea showed that HMT but not DAO decreases bronchoconstrictor responses to histamine and antigen challenge.[6,7] However, it remains unknown which enzyme is responsible for the degradation of histamine in the human airway and where the enzyme is located. Therefore, human bronchial strips were studied under isometric conditions *in vitro* to elucidate the modulatory role of histamine-degrading enzymes in the histamine-mediated bronchoconstriction.

### Materials and Methods

*Preparation of tissues:* Human lung tissues were obtained from 23 patients at thoracotomies performed because of carcinoma. After surgical removal, pieces of macroscopically normal lung tissues were rapidly immersed in Krebs–Henseleit solution consisting of the following composition (in mM): NaCl, 118; KCl, 5.9; $MgSO_4$, 1.2; $CaCl_2$, 2.5; $NaH_2PO_4$, 1.2; $NaHCO_3$, 25.5; and D-glucose, 5.6; gassed with a mixture of 95% $O_2$–5% $CO_2$ at 37°C. Cartilaginous bronchi, 2 to 4 mm in internal diameter, were then dissected free of parenchyma, fat and surrounding connective tissues, and cut helically at a 45° pitch to obtain bronchial strips measuring 2 to 3 mm in width and approximately 20 mm in length. Between two and eight strips were dissected from each specimen and mounted in 14 ml organ chambers containing Krebs–Henseleit solution aerated with 95% $O_2$–5% $CO_2$ at 37°C (pH 7.4, $P_{CO_2}$ 38 Torr, $P_{O_2} > 500$ Torr). Contractile responses were continuously measured isometrically with a force–displacement transducer (Nihon Kohden, JB-652T, Tokyo, Japan) and were recorded on a pen recorder (Nihon Kohden, WT-685G). The tissues were allowed to equilibrate for 60 min while they were washed with Krebs–Henseleit solution every 15 min, and the resting tension was adjusted to 1 g. A contractile response was determined as the difference between peak tension developed and resting tension. All experiments were conducted in the presence of indomethacin ($3 \times 10^{-6}$ M) and ranitidine ($6 \times 10^{-5}$ M) to avoid prostaglandin release and histamine tachyphylaxis,[8] respectively.

*Effects of histamine-degrading enzyme inhibitors:* Following the equilibration period, histamine was added to the chamber in a cumulative manner at concentrations ranging from $10^{-8}$ to $10^{-3}$ M in half-molar increments at 5 min intervals or 2 min after

© 1994 Rapid Communications of Oxford Ltd stable plateau was achieved, whichever was the longer period. After establishing the first concentration–response curves, tissues were washed with Krebs–Henseleit solution until the tension returned to the baseline level and, 60 min later, SKF 91488 ($10^{-4}$ M), an inhibitor of HMT,[9,10] or aminoguanidine ($10^{-4}$ M), an inhibitor of DAO,[11] was added. Twenty min later, the second concentration–response curves for histamine were generated in a similar manner. In a control experiment, two successive concentration–response curves for histamine were likewise generated without addition of an inhibitor.

To test whether the inhibition of HMT activity also alters the contractile responses to other spasmogenic agonists, the effect of SKF 91488 ($10^{-4}$ M) on the concentration–response curves for acetylcholine and KCl were examined, with the same time sequence for the contractile responses to histamine.

At the end of these experiments, each bronchial strip was blotted on a gauze pad and weighed. Active tensions were normalized for tissue weight and expressed as grams tension per gram of tissue weight. To characterize the concentration–response curves, the maximal contractile response ($E_{max}$) and the negative logarithm of molar concentration of agonist required to produce 50% of $E_{max}$ ($pD_2$) were determined by linear regression analysis.

To assess concentration-dependent effects of histamine-degrading enzyme inhibitors, contractile responses of bronchial strips to $10^{-5}$ M histamine were determined in the absence and presence of either SKF 91488 or aminoguanidine ($10^{-8}$ to $10^{-3}$ M). In this experiment, after determining the first responses to histamine, tissues were washed, applied with a single concentration of either inhibitor and, 20 min later, the second responses to histamine were determined.

To assess whether the modulation of the contractile responses to histamine by SKF 91488 was associated with the epithelium, concentration–response curves for histamine were constructed in the absence and presence of SKF 91488 ($10^{-4}$ M) in tissues with the epithelial cells denuded. Bronchial strips were gently rubbed off their luminal surface with a moist cotton swab, and confirmation of the successful removal of the epithelium was histologically performed in randomly selected tissues.

*Measurement of HMT activity:* The activity of HMT was measured in bronchial tissues with and without epithelium according to the method by Fukuda *et al.*[12] Briefly, excised bronchi were homogenized in a glass homogenizer, with four volumes of ice-cold phosphate buffered saline (0.05 M, pH 7.4) containing 1 mM dithiothreitol and 1% polyethyleneglycol. The homogenate was centrifuged at 4°C (120 000 × **g**, 1 h), and the supernatant was dialysed three times for 8 h against 100 volumes of the buffer. The reaction of HMT was carried out at 37°C in 0.5 ml of a mixture of 0.1 ml of the supernatant, 0.3 ml of 0.1 M phosphate buffered saline containing 0.1 mM pargyline and 0.1 mM aminoguanidine (pH 7.4), 0.05 ml of 1 mM histamine and 0.05 ml of S-adenosyl-L-methionine. After incubation, $N^{\tau}$-methylhistamine was separated from histamine by high-performance liquid chromatography on a weak cation exchanger (Toyo-Soda Co., TSKgel CM2SW, Tokyo), with 37.5 mM citric acid, 1.25% imidazole and 20% acetonitrile, with the mobile phase at a flow rate of 1.0 ml/min. The fluorescence intensity of the reaction mixture was then measured using a post-column derivatization with *o*-phthalaldehyde and 2-mercaptoethanol, and the HMT activity was expressed as pmol of $N^{\tau}$-methylhistamine formed per h per mg of protein as determined by the method of Lowry *et al.*[13]

*Drugs:* The following drugs were used: histamine diphosphate, indomethacin, acetylcholine chloride, KCl, aminoguanidine (Sigma Chemical Co., St Louis, MO); and ranitidine (Glaxo Co., Tokyo). SKF 91488 (S-[4[(N,N-dimethylamino)-butyl] isothiourea) was a gift from Smith Kline Co. (Philadelphia, PA).

*Statistics:* All values were expressed as means ± S.E. Comparative statistical analysis was performed using ANOVA followed by either Tukey's test for multiple comparisons or by Student's *t*-test; $n$ refers to the number of preparations, and $p < 0.05$ was considered statistically significant.

### Results

*Muscle contraction:* As demonstrated in Fig. 1, in the control experiment both the $E_{max}$ and the $pD_2$ values of the second histamine concentration–response curves of human bronchial strips were not significantly different from those of the first concentration–response curves. Thus, the histamine-induced tachyphylaxis was not observed in the presence of indomethacin and ranitidine. Addition of SKF 91488 ($10^{-4}$ M) and aminoguanidine ($10^{-4}$ M) did not alter the resting tension. Pretreatment of tissues with SKF 91488 potentiated the contractile responses to histamine, causing a leftward displacement of the histamine concentration–response curves with the $pD_2$ values being increased from 5.0 ± 0.1 to 5.8 ± 0.3 ($p < 0.01$, $n = 10$) but the $E_{max}$ values remained unchanged. On the other hand, aminoguanidine also tended to potentiate the contractile responses to histamine, but the change in the $pD_2$ values did not reach a significant level (5.0 ± 0.2 to 5.2 ± 0.3, $p < 0.05$, $n = 9$).

The SKF 91488-induced potentiation of the contractile responses to $10^{-5}$ M histamine was concentration dependent, with a threshold concentration and the maximal increase from the baseline value being

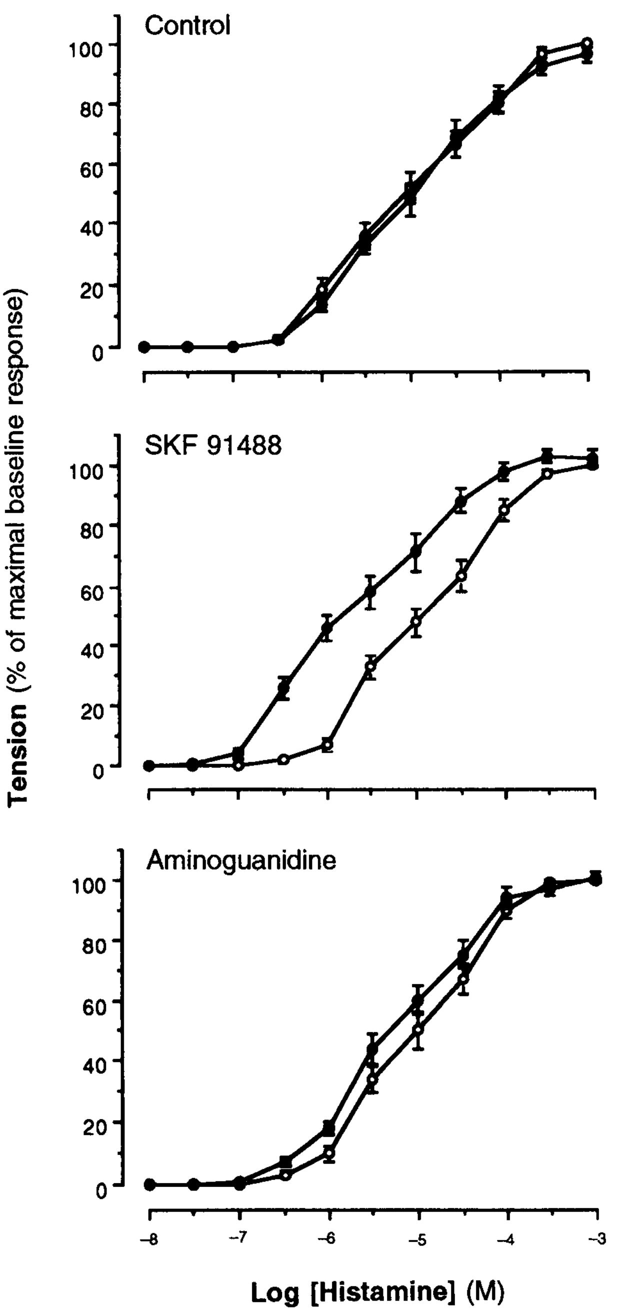

FIG. 1. Effects of SKF 91488 ($10^{-4}$ M) and aminoguanidine ($10^{-4}$ M) on contractile responses of human bronchial strips to histamine. After establishing baseline responses to histamine (*open circles*), tissues were washed, either inhibitor was added and, 20 min later, the second concentration–response curves were generated (*closed circles*). Tissues without addition of an inhibitor served as control experiments. Values are expressed as percentage of the maximal baseline response. Each point represents mean ± S.E.; $n = 10$ for control and SKF 91488, and $n = 9$ for aminoguanidine.

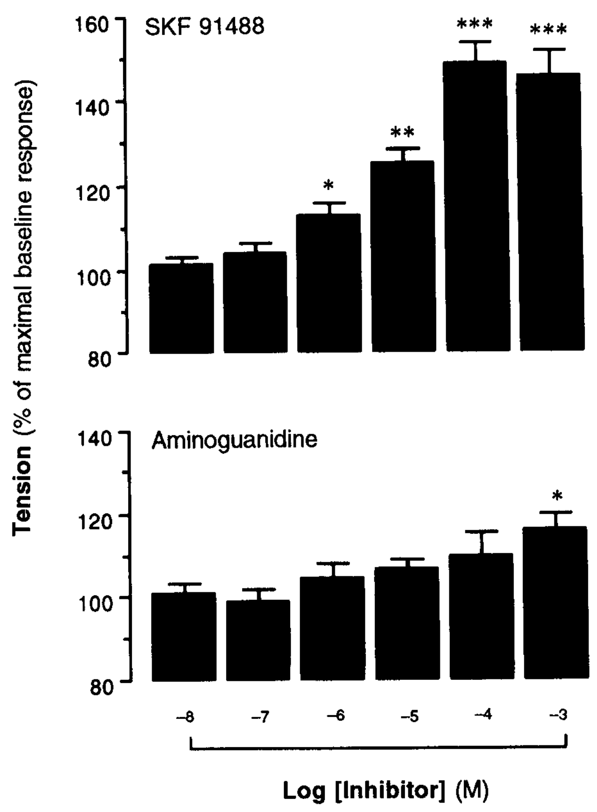

FIG. 2. Concentration dependent effects of SKF 91488 and aminoguanidine on contractile responses to $10^{-5}$ M histamine. Values are expressed as percent of the baseline responses obtained before the addition of an inhibitor. Data are means ± S.E.; $n = 8$ for each column. *$p < 0.05$, **$p < 0.01$, ***$p < 0.001$, significantly different from baseline values.

**Table 1.** Effect of SKF 91488 on contractile responses of human bronchail strips

| | $pD_2$ | | $E_{max}$ | | |
|---|---|---|---|---|---|
| Agonist | Control | SKF 91488 ($10^{-4}$ M) | Control | SKF 91488 ($10^{-4}$ M) | CR |
| Histamine | 5.0 ± 0.1 | 5.8 ± 0.3** | 13.2 ± 2.2 | 13.8 ± 3.1 | 6.3 |
| Acetylcholine | 6.2 ± 0.2 | 6.4 ± 0.3 | 11.8 ± 1.9 | 12.6 ± 2.0 | 1.6 |
| KCl | 1.9 ± 0.1 | 1.9 ± 0.2 | 10.7 ± 1.9 | 11.5 ± 2.6 | 1.0 |

$pD_2$, negative logarithm of molar concentration of the agonist required to produce a half-maximal effect; $E_{max}$, maximal contraction as expressed by g/g tissue; CR, concentration ratio as determined by antilog ($pD_2$ in the presence of SKF 91488 – control $pD_2$). Data are means ± S.E.; $n = 10$ for histamine and acetylcholine and $n = 11$ for KCl. ** $p < 0.01$, significantly different from control values.

$10^{-6}$ M and 48.3 ± 4.9% ($p < 0.001$, $n = 8$), respectively, whereas aminoguanidine caused a significant increase only at $10^{-3}$ M by 12.5 ± 3.1% ($p < 0.05$, $n = 8$, Fig. 2).

In contrast to the effect on histamine-induced contraction, pretreatment of tissues with SKF 91488 ($10^{-4}$ M) had no effect on the contractile responses to other spasmogenic agonists, acetylcholine and KCl (Table 1).

In bronchial strips with the epithelium removed, SKF 91488 ($10^{-4}$ M) still potentiated the contractile responses to histamine, the $pD_2$ values being increased from 5.6 ± 0.3 to 6.1 ± 0.2 ($p < 0.05$, $n = 11$, Fig. 3). However, the magnitude of the leftward shift

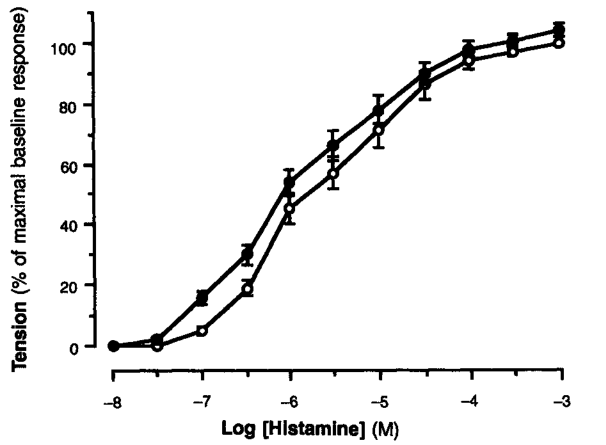

FIG. 3. Effect of SKF 91488 ($10^{-4}$ M) on contractile responses to histamine in bronchial strips with the epithelium removed. After establishing baseline responses to histamine (*open circles*), SKF 91488 was added and the second concentration–response curves were generated (*closed circles*). Values are expressed as percent of the maximal baseline response. Each point represents mean ± S.E.; $n = 11$.

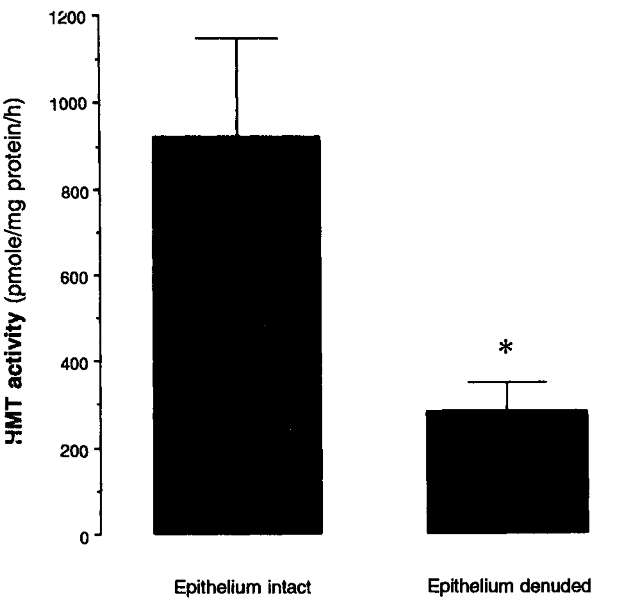

FIG. 4. Histamine N-methyltransferase (HMT) activity in human bronchial tissues. Activity of the enzyme was measured by high-performance liquid chromatography based on post-column derivatization with *o*-phthalaldehyde. Data are means ± S.E.; $n = 11$ for epithelium-intact tissues and $n = 12$ for epithelium-denuded tissues. *$p < 0.05$, significantly different from values for epithelium-intact tissues.

of the concentration–response curves was small compared with the epithelium-intact tissues; SKF 91488 potentiated histamine-induced contraction by 6.3-fold in epithelium-intact strips and by 3.2-fold in epithelium-denuded tissues.

*Tissue HMT activity:* The activity of HMT in human bronchi determined by high-performance liquid chromatography was 920 ± 230 pmol/mg protein/h ($n = 11$) for the epithelium-intact tissues and 280 ± 70 pmol/mg protein/h ($n = 12$) for the tissues without epithelium. There was a significant difference between these values ($p < 0.05$, Fig. 4).

### Discussion

The present *in vitro* studies demonstrate that HMT probably localized to human airway epithelium may play a protective role in the histamine-mediated bronchoconstriction through the degradation of histamine to its inactive metabolite. This notion is based on the following findings. First, pretreatment of bronchial strips with the HMT inhibitor SKF 91488[9,10] potentiated the histamine-induced contraction in a concentration dependent fashion without affecting contractile responses to other spasmogenic agonists including acetylcholine and KCl; secondly, the magnitude of the SKF-induced leftward displacement of histamine concentration–response curves was significantly less in bronchial strips with the epithelium mechanically removed than in the epithelium-intact tissues; and thirdly, the activity of HMT was measured by high-performance liquid chromatography using post-column derivatization with *o*-phthalaldehyde and it was found that the HMT activity of human bronchial tissues was greatly decreased by removal of the epithelium.

It has been known that histamine is metabolized by two major enzymes, HMT and DAO, located on a variety of mammalian tissues and inflammatory cells.[14] HMT catalyses methyl transfer from S-adenosyl-L-methionine to histamine to form N-methylhistamine, which is further metabolized by monoamine oxidase to N-methylimidazole acetic acid,[15] and DAO also metabolizes histamine to N-methylimidazole acetic acid.[16] In the present study, it was found that, in contrast to the effect of SKF 91488, aminoguanidine at concentrations sufficient to inhibit DAO activity[11] had little effect on the contractile responses to histamine. Therefore, histamine may be metabolized principally through the HMT pathway in human airways, as is also true in the brain.[17]

The airway epithelium has been shown to inhibit bronchoconstrictor responses to a variety of stimuli by releasing epithelium-derived relaxing factor, which is not a product of arachidonic acid and is not nitric oxide,[18–20] and by metabolizing tachykinins with neutral endopeptidase.[21] Concerning the histamine metabolism, there are conflicting reports on guinea-pig tracheal epithelium. Lindström *et al.*[22] showed the aminoguanidine-induced potentiation of the contractile responses to histamine, but Ohrui *et al.*[6] recently reported the lack of aminoguanidine's effect and showed the presence of HMT activity and its mRNA in the epithelium by *in situ* hybridization, and Sekizawa *et al.*[7] reported a similar finding in antigen-induced bronchoconstriction *in vivo*. The present findings on human bronchi were in agreement with the latter two reports. Although DAO activity was not measured, the results of the muscle bath experiments suggest that the epithelial HMT activity is more important than DAO in limiting the biological actions of histamine in human airways. In tissues without epithelium, the HMT activity was still present and the SKF 91488-induced potentiation of the contractile responses to histamine was small but still significant. These findings suggest that HMT localized to other cell types such as endothelial cells[6] could also contribute to histamine degradation.

In conclusion, the histamine-degrading enzyme HMT in the airway epithelium may play a modulatory role in the bronchoconstriction in human airways through a metabolism of histamine. The authors therefore speculate that airway hyperresponsiveness to histamine in asthmatic subjects could be due, at least in part, to the epithelial damage-associated loss of HMT activity.

### References

1. Braude S, Royston D, Coe C, Barnes PJ. Histamine increases lung permeability by an $H_2$-receptor mechanism. *Lancet* 1984; **2**: 372–374.
2. Shelhamer JH, Marom Z, Kaliner M. Immunologic and neuropharmacologic stimulation of mucous glycoprotein release from human airways *in vitro*. *J Clin Invest* 1980; **66**: 1400–1408.
3. Scadding JG. Definition and clinical categories of asthma. In: Clark TJH, Godfrey S, eds. *Asthma*. London: Chapman Hall, 1983; 1–11.
4. Beavens MA. Histamine: its role in physiologic and pathologic process. *Monogr Allergy* 1978; **13**: 1–20.
5. Zeinger RS, Yurdin DL, Colten HR. Histamine metabolism. II. Cellular and subcellular localization of the catabolic enzymes, histaminase and histamine methyltransferase in human leukocytes. *J Allergy Clin Immunol* 1976; **58**: 172–179.
6. Ohrui T, Yamauchi K, Sekizawa K, *et al.* Histamine N-methyltransferase controls the contractile responses of guinea pig trachea to histamine. *J Pharmacol Exp Ther* 1992; **261**: 1268–1272.
7. Sekizawa K, Nakazawa H, Ohrui T, *et al.* Histamine N-methyltransferase modulates histamine- and antigen-induced bronchoconstriction in guinea pigs *in vivo*. *Am Rev Respir Dis* 1993; **147**: 92–96.
8. Knight DA, Stewart GA, Thompson PJ. Histamine tachyphylaxis in human airway smooth muscle: the role of $H_2$-receptors and the bronchial epithelium. *Am Rev Respir Dis* 1992; **146**: 137–140.
9. Beaven MA, Shaff RE. New inhibitors of histamine N-methyltransferase. *Biochem Pharmacol* 1979; **28**: 183–188.
10. Beaven MA, Shaff RE. Inhibition of histamine methylation *in vivo* by the Dimaprit analog, SKF compound 91488. *Agents Actions* 1979; **9**: 455–460.
11. Schuler W. Zur Hemmung der Deaminooxydase (Histaminase). *Experientia* 1952; **8**: 230–232.
12. Fukuda H, Yamatodani A, Imamura I. High-performance liquid chromatographic determination of histamine N-methyltransferase activity. *J Chromatogr* 1991; **567**: 459–464.
13. Lowry OH, Rosenbrough NJ, Farr AL, Randall RJ. Protein measurement with the Folin phenol reagent. *J Biol Chem* 1951; **193**: 265–275.
14. White MV, Slater JE, Kaliner MA. Histamine and asthma. *Am Rev Respir Dis* 1987; **135**: 1165–1176.
15. Schayer RW, Karjala SA. Ring N methylation: a major route of histamine metabolism. *J Biol Chem* 1956; **221**: 307–313.
16. Douglas WW. Histamine and 5-hydroxytryptamine (serotonin) and their antagonists. In: Gilman AG, Goodman LS, Rall TW, Murad F, eds. *The Pharmacological Basis of Therapeutics*. New York: Macmillan Publishing Co., 1985; 605–638.
17. Schwartz JC. Histaminergic mechanisms in brain. *Ann Rev Pharmacol Toxicol* 1977; **17**: 325–339.
18. Flavahan NA, Aarhus LL, Rimele TJ, Vanhoutte PM. Respiratory epithelium inhibits bronchial smooth muscle tone. *J Appl Physiol* 1985; **58**: 834–838.
19. Shikano K, Ohlstein EH, Berkowitz BA. Differential activity of epithelium-derived relaxing factor and nitric oxide in smooth muscle. *Br J Pharmacol* 1987; **92**: 483–485.
20. Munakata M, Masaki Y, Sakuma I, *et al.* Pharmacological differentiation of epithelium-derived relaxing factor from nitric oxide. *J Appl Physiol* 1990; **69**: 665–670.
21. Sekizawa K, Tamaoki J, Graf PD, Basbaum CB, Borson DB, Nadel JA. Enkephalinase inhibitor potentiates mammalian tachykinin-induced contraction in ferret trachea. *J Pharmacol Exp Ther* 1987; **243**: 1211–1217.
22. Lindström EG, Andersson RGG, Granérus G, Grundström N. Is the airway epithelium responsible for histamine metabolism in the trachea of guinea pig? *Agents Actions* 1991; **33**: 170–172.

ACKNOWLEDGEMENTS: The authors thank Smith Kline Co. for providing us with SKF 91488. This work was supported in part by a grant No. 04670476 from the Ministry of Education, Science and Culture, Japan.

**Received 4 October 1993;**
**accepted in revised form 20 December 1993**
